# Preferences of melanoma patients to accept adjuvant therapy and toxicity – a qualitative substudy of the GerMelaTox-A project

**DOI:** 10.1007/s00432-024-06014-8

**Published:** 2024-12-04

**Authors:** Toni Maria Janke, Laura Moysig, Christine Blome, Katharina C. Kähler

**Affiliations:** 1 Institute for Health Services Research in Dermatology and Nursing (IVDP), University Medical Center Hamburg-Eppendorf (UKE), Hamburg, Germany Martinistraße 52, 20246; 2 Department of Dermatology, University Hospital Schleswig-Holstein (UKSH), Campus Kiel, Kiel, Germany

**Keywords:** Adjuvant therapy, Patient preferences, Qualitative interviews, Melanoma

## Abstract

**Purpose:**

Targeted treatment and immunotherapy, both adjuvant treatment options, come with a certain toxicity and can cause severe side effects. To date, data about the underlying reasons for patients to accept or reject specific types of adjuvant therapy is scarce. Therefore, this study investigates the motives of melanoma patients for tolerating or rejecting adjuvant therapy and its side effects.

**Methods:**

We conducted semi-structured interviews with a subsample of patients to investigate the underlying reasons for treatment decisions in a quantitative treatment-trade off study. Categorisation was conducted using qualitative content analysis.

**Results:**

The 17 participants had a mean age of 55.5 years and 12 were female. The final category system covered three clusters. The cluster “type of therapy and therapy process” described therapy-related aspects that affect acceptability of adjuvant treatments. Prospect of treatment benefit and side effects were important aspects. Route of administration and physician visits should be convenient. The cluster “way of living” described the influence that activities and circumstances of life organisation have on acceptability. Participants wished treatment to affect everyday life as little as possible. Maintaining sufficient quality of life was mentioned to be crucial. The cluster “emotions and feelings” described optimism and hope but also mental strain originating from possible treatment options.

**Conclusion:**

Patients in our study indicate high willingness to undergo adjuvant therapy, even when facing toxicity. The evaluation of potential side effects and prospects of treatment benefit is highly individual. Therefore, it is important to consider personal patient preferences to make appropriate and shared decision-making.

## Introduction

After complete resection of malignant tumours, adjuvant therapy can be considered a supportive measure to reduce the chance of recurrence or metastatic diseases and hence improve disease-free survival (DFS) and overall survival (OS) (Long et al. 2022). After targeted therapies (TT) and immunotherapies have dramatically improved survival in patients with advanced melanoma (Garutti et al. 2022), these forms of therapy have been transferred also to the adjuvant setting. TT and immunotherapy can be beneficial especially for patients in stage II, III and IV according to the American Joint Committee on Cancer (AJCC) being at high risk for recurrence or death from melanoma. In TT, patients receive orally BRAF and MEK inhibitors. Immunotherapy can be applied with a single medication (PD1 antibody; ICI) or with two medications (PD1 antibody and CTLA4 antibody; cICI), both applied as infusions. TT and (c)ICI show significant improvements in patients DFS and OS and are increasingly used in routine care (Kobeissi and Tarhini 2022; Livingstone et al. 2022).

Treatment with TT may cause side effects such as fever, joint paint, gastrointestinal complaints or eye disorders (Lazaroff and Bolotin 2023). Whereas toxicity of (c)ICI has the potential to induce autoimmune side effects in various organs, which might become chronic (Schulz et al. 2022) or even be lethal in a small number of patients (Wang et al. 2018). For those patients with severe side effects health-related quality of life (HRQoL) may be impaired persistently, which might lead to treatment cessation (Wang et al. 2018; Pedersen et al. 2023). However, in most patients receiving (c)ICI, HRQoL is not impaired or only in the short-term (Bottomley et al. 2021; Khattak et al. 2022; Pedersen et al. 2023).

In addition to clinical recommendations, patients might consider very individual aspects to be relevant for the final treatment decision. Previous studies on patient preferences mostly used quantitative methods, such as discrete choice experiments, (e.g., (Beusterien et al. 2017; Stenehjem et al. 2019) and often investigated the preferences regarding interferon therapy (e.g., Kerry L. Kilbridge et al.; Kähler et al. 2016), which in practice has been largely replaced by the newer therapies with TT and (c)ICI. For all types of treatment, studies suggest that patients would be willing to accept (severe) side effects for a significant reduction of the risk of recurrence (Kähler et al. 2016; Stellato et al. 2019). However, data about the underlying reasons for accepting or rejecting specific form of treatment is scarce (Liu et al. 2019; Livingstone et al. 2020). A deeper understanding of these underlying reasons could help guiding decision making in clinical practice.

Therefore, the research question of the present substudy of the GerMelaTox-A project (English translation of the full project title: “Preferences of German and Swiss melanoma patients for toxicities versus melanoma recurrence during adjuvant treatment”; (Kähler et al. 2023) is “What are personal motives for tolerating or rejecting adjuvant therapy and its side effects?”. The main study quantitatively assessed the attitudes and preferences of melanoma patients for toxicity in adjuvant treatment with TT and (c)ICI. In the present qualitative substudy, we aimed to understand patients’ perspectives, attitudes and judgements about specific aspects of different forms of adjuvant treatment.

## Methods

### The main study

The GerMelaTox-A study was conducted in eleven skin cancer centres in Germany and Switzerland between June 2019 and January 2022. It included patients above the age of 18 years having experienced a melanoma diagnosis. To avoid ethical conflict, only low-risk melanoma patients were included, defined as being on a T1a level, without sentinel node biopsy or significant co-morbidities. Participants completed a quantitative questionnaire using the treatment-trade-off (TTO) approach, in which they were asked to imagine a melanoma with a 30% chance of DFS and a 50% chance of OS after 5 years. In total, twelve scenarios were presented of which eleven described the three treatment opportunities (TT; ICI; cICI) with different degrees of side effects (no; mild to moderate; severe) and impacts on blood counts (no impact; abnormal values) and one scenario describing a recurrent melanoma after adjuvant therapy. For each scenario, patients were supposed to state the minimum number of prevented recurrences and prevented deaths (i.e., treatment effectiveness), that they would consider sufficient for them to accept this treatment. Patients also rated their overall preference for each scenario using a visual analogue scale (VAS). This study was conducted according to the principles of the Declaration of Helsinki and ethically approved by the Ethics Committee of Christian-Albrechts-University Kiel (D 435/20).

## Participants

For the present qualitative study, a subsample was recruited. These were patients from one of the German study centres (University Hospital Schleswig-Holstein, Campus Kiel). We targeted to recruit a heterogeneous sample regarding sex, age, and responses in the quantitative questionnaire. Sample size was not determined a priori, as is common in qualitative research. Instead, patients were recruited until saturation was achieved, which means that additional interviews had resulted in no new categories. It was assumed that this would be reached after approximately 20 interviews. Participants received written and verbal information about the study and signed informed consent.

## Data assessment and analysis

From September to November 2020, semi-structured interviews were conducted timely after participants had completed the quantitative questionnaire of the main study. The interviews were conducted by one of the authors (LM), who had neither personal relationship with the interviewees nor was involved in their medical care. While the first two interviews took place in person, subsequent interviews were conducted via phone due to the COVID-19 pandemic.

The interview guideline was developed by the interviewer (LM) and discussed with two qualitatively experienced researchers (TMJ, CB) taking into account the quantitative questionnaire and the qualitative research question. First, the interviewer introduced the research project and asked the participants about their experiences when answering the quantitative questionnaire. Then, as a superordinate question, the interviewer asked about aspects (e.g., priorities, side-effects) influencing the participant’s decisions when answering the questionnaire. Following this, the interviewer briefly summarised the different treatment options and went through the twelve scenarios. For each scenario, the interviewer asked how the participant had come to the final decision on DFS, OS and VAS and what the reasons were. After this, the interviewer explicitly asked participants in how far the number of physician visits, blood samplings and infusions in the scenarios had influenced their responses. Finally, the interviewer asked which treatment the participant would choose and whether and why he or she would ask other people for their advice in decision-making. Due to the character of the semi-structured interview, it was possible to address topics newly raised by the participant or to change the order of questions when the interviewer assumed this to be necessary based on the course of conversation.

All interviews were audio recorded and transcribed verbatim. Interviews were then categorized using qualitative structuring content analysis according to Mayring (2010) as it can be assumed that patients’ statements offer a direct insight into their thoughts and judgements. After familiarisation with the data, one author (LM) analysed the first interview by paraphrasing relevant content-bearing text passages, which were then categorized. The category system enlarged with the categorization of subsequent interviews. It was frequently discussed with two other authors (TMJ, CB) experienced in qualitative research and further higher hierarchical categories were developed. The main categories were subsumed into four different clusters. When additional interviews did not result in further subcategories, data saturation was assumed to be reached and data assessment thus closed.

## Results

All 17 participants of the quantitative study who were approached for the qualitative substudy agreed to participate. They had a mean age of 55.5 years (range: 35–76 years); 12 were female and 5 male. Three persons had been diagnosed with further tumours beyond the malign melanoma and 14 reported having experienced a history of tumour of a close friend or relative. The interviews lasted on average 64 min (range: 43–95 min).

The final category system was divided into four clusters: type of therapy and therapy process; way of living; emotions and feelings; methodological issues.

## Type of therapy and therapy process

**Table 1:** Cluster Type of therapy and therapy process

Main category	Subcategory	Quote	#
**Side effects**	Impact side effect severities on tolerability	“But well, the side effects, I say, you described the different scenarios in your questionnaire and I think it’s already starting to get unpleasant with the moderate side effects. […] If you decide in favour of this therapy, then you shoulder it, but it’s no fun.” (P14, male, 61 years)	Q1.1
	Specific side effects	“Yes, that’s the joint pain, for example, because that can be very bad for me. (…) My neuropathy, that lasted a long time, I also had severe pain, but mainly in my legs, and that’s what I meant now. It can be quite unpleasant.“ (P2, female, 76 years)	Q1.2
	Aspects of side effects	“So it becomes more difficult if yes, well, if you then have constant vomiting and frequent nausea, these usual side effects that are in every package leaflet, and that’s not one in 10,000, but one in ten patients has these side effects.“ (P16, male, 35 years)	Q1.3
	Medication	“So I would rather endure pain than swallow the drug.“ (P1, female, 63 years)	Q1.4
	Altered blood counts	“Yes, that there are additional side effects, such as the blood values; bad blood values are always a risk. Whether it’s for the heart always depends on which blood values are bad, or the liver. That caus-es other diseases that you can get as a result, secondary diseases. Therefore, the one less than the other.” (P11, female, 51 years)	Q1.5
**Route of administration**	Being convenient and easy to integrate	“Yes, because I think that’s the thing, I don’t think taking a tablet is so bad, of course you have to remember the time, but it’s a quick and easy form of therapy, I say. And if you don’t have to rest too much and can get on with your life, then I think that’s ok.“ (P13, female, 51 years)	Q1.6
		“Yes, it’s simply based on the fact that it’s more bearable for me to have an infusion, to keep these fixed appointments, than to take them, where, as I said, you’re one hundred per cent restricted. I can simply keep appointments better than taking these tablets. And that’s why I thought that this infusion therapy would be easier for me to keep […]“ (P11, female, 51 years)	Q1.7
	Responsibility and control	“Well, I think it would be important for me to be in contact with the doctor treating me, and as convenient as it may be with the tablets, I would have some concerns if I were to take tablets myself and then perhaps come to the doctor’s office less often. So I think a regular check-up like this would be a safety measure for me.“ (P8, female, 53 years)	Q1.8
	Experience of and association with disease	“No, but of course I have the problem that taking blood doesn’t always work so smoothly because I have rolling veins, so it can be quite unpleasant. When they stick the needle in five times, it’s no fun. And as I said, of course the whole thing is terribly serious and awful, but if you ask about differences, what do I find better or what do I find worse, then these are factors where I say it’s better if I don’t need all that stuff then.“ (P14, male, 61 years)	Q1.9
		“Well, let me put it this way, it’s always the case that you sit there in a room and then get it connected, now it happens like that, I myself accompanied a friend of mine who unfortunately passed away two years ago and who also always had her chemo. And when you’re sitting there like that, it’s more emotionally stronger, it’s more serious. The illness is closer, I say, or yes.” (P13, female, 51 years)	Q1.10
	No impact	“No, so whether you take tablets now or whether you get it somehow via infusion, that wouldn’t be the difference for me now“ (P7, female, 37 years)	Q1.11
		“If I’m there anyway and I get an infusion and my blood is taken at the same appointment, that wouldn’t affect me at all.“ (P3, female, 59 years)	Q1.12
**Visits to physician**	No impact	“No, no, I think that’s something you can plan for. Or something I can plan for. That wouldn’t bother me at all.“ (P7, female, 37 years)	Q1.13
	Reassuring effect	“Yes, but that’s not a bad thing. If there’s a check-up, it’s only good for you and then you have a bit more security if they always look at the blood count and (pause) I think you’re somehow in good hands.“ (P1, female, 63 years)	Q1.14
	Impact on everyday life	„Yes, that’s the only thing for me in this scenario, that you are of course a little restricted in your free life planning when it comes to medical examinations and appointments. (…) For me, it’s just a small restriction (laughs) in terms of quality of life, that you are determined by others, that you have other plans, I say.“ (P10, male, 56 years)	Q1.15
	Presence of disease	“And of course, the more visits to the doctor and the more hospitalisations and the more infusions and the more you do […] the more the problem that you then want to combat moves to the forefront of your life, the less beautiful the whole thing is. […] In other words, the whole course of life and so on is determining, which is of course also necessary for survival to take place at all, but the further it goes, the more sustainably the whole way of life is influenced by it.” (P14, male, 61 years)	Q1.16
**Prospect of treatment benefit**	Prospect	“Yes, I just thought to myself that this is a great physical strain, a great physical and psychological strain. And then I actually expect it to be more effective. […] So then, if you can just say to me now: ‘ok, you have to take on a lot, but the chance that you’ll survive the whole thing is only ten or twenty per cent anyway’, then you have to weigh up for yourself, do I take it on myself now or do I accept that I’ll die from it.” (P9, female, 48 years)	Q1.17
	Consequences	“Yes, of course you have to, if these other things haven’t helped. You don’t want to get straight into the full blatant, which also exists, you want to endure what your body can, then you should accept that. (…) Yes, with chemo and so on, that’s the worst, where the healing is perhaps good, but a bit more extreme than this thing now.” (P17, male, 47 years)	Q1.18
	Risk of recurrence and the risk of dying	“I did, it was 50 of them from the relapse or death, I didn’t differentiate that much. I saw it in a similar way.” (P16, male, 35 years)	Q1.19
		“Yes, I always think that way about the relapse, so I assume that this is the case with the relapse. Then it doesn’t mean that even if people relapse, it’s that bad again. So that it’s really bad again. So that’s something else for me than when I say, or imagine, how many people would die from it, so actually dying.” (P7, female, 37 years)	Q1.20
**Source of information and decision aid**	Physicians	“I think so. I would first seek advice from my treating doctor. And if I trusted him, I would, I think I would accept the way he suggests the therapy or maybe I would research what is suggested on the internet.” (P12, female, 72 years)	Q1.21
	Second opinion	“So, as I said, I would definitely get a second opinion from the doctor treating me and my dermatologist, of course, but I wouldn’t mhm (…) ask around (…) with people who are affected, perhaps.“ (P5, female, 52 years)	Q1.22
	Exchange	“Yes, I do believe that. I have a really good circle of friends. But I think especially for things like that, it’s important to talk about it with someone who is affected or with a, I don’t know, a self-help group or whatever in that direction, even a neutral person in inverted commas, just talk about it then. Or with people who have also been affected, because if you haven’t experienced such things, you can’t really empathise with them. […] I don’t want pity or anything like that, just sympathy and exchange on that level or just understanding somewhere. And perhaps giving each other a bit of strength, that would be important to me.” (P15, female, 61 years)	Q1.23
	Independence	“And then I also have medically knowledgeable people in my circle of acquaintances and I would also find out for myself. You can acquire a lot of knowledge in a short space of time. And I would pull out all the stops before saying don’t do one or the other. So I’d say you can get a lot of impressions in a very short time and learn a lot.” (P14, male, 61 years)	Q1.24
	Psychological support and counselling	“Afterwards I realised (…) that I always think psychological care is also so important (…) but I always realise that this is often a weak point […] the doctors are pretty straightforward […] so it’s purely medical, but what it does to you personally, I think you’re a bit alone with that. And if you don’t help your-self, then of course you’re on your own, I think.“ (P13, female, 51 years)	Q1.25
**Alternative therapy methods**	Less invasive options	“That’s, it’s simply a prophylactic event and the restrictions are already quite enormous (…) how can I protect myself from it. Very close dermatological care, (…) a change in diet, strengthening the immune system and, and, and. Before you then resort to conventional medicine, with correspondingly serious, really lasting side effects.“ (P14, m male 61 years)	Q1.26
	No alternative	“If that was the only option that could be offered to me at all, then it would be perfectly fine for me to try it, even if I felt side effects for a year.” (P8, female, 53 years)	Q1.27

The cluster “type of therapy and therapy process” (Table 1) describes therapy-related aspects that affect the acceptability of adjuvant treatments. This cluster was the largest in term of quantity of references in the interviews, which was to be expected based on the interview guideline. Regarding **side effects**, different severities were presented in the scenarios. The patients discussed ***impact of side effect severities on the tolerability*** in all interviews. Mostly, scenarios without side effects were acceptable for the participants, even though one person expected side effects to occur anyways due to bad experiences with previous side effect profiles of medication. Mild to moderate side effects were evaluated differently from no impact on acceptability to reduced acceptability. Despite reduced acceptability, most patients stated that they would still tolerate these scenarios (Q1.1), also because they compared side effects to potential risks of cancer, such as increased mortality. In contrast, severe side effects often led to significantly reduced acceptability as the impact of these side effects would cause too much suffering. Some patients stated to prefer living without treatment compared to living with these impairments of severe side effects. Others stated that they would consider these treatments only as last resort after trying to live without. Some patients underlined ***specific side effects*** that they would perceive as particularly burdensome. This included gastro-intestinal problems as well as tiredness, weakness and being downhearted, which patients often associated with each other. Additionally, vomiting, skin problems, headaches, joint pain, weight loss, cardiovascular problems, symptoms of cold and flu as well as fever and chills were mentioned as burdensome due to their impact on patients’ daily life and quality of life (Q1.2). In contrast, others considered symptoms of cold and flu, skin problems and diarrhoea as acceptable as these would be well-manageable. Some patients stated that the combined appearance of different side effects would reduce the acceptability. Participants also considered specific ***aspects of side effects*** as decisive. This included the probability of the occurrence and the frequency and duration of the side effects (Q1.3). Side effects were rated as less tolerable when they caused physical impairments, such as damage to organs lasting beyond the adjuvant treatment. Also, some patients did not believe that such treatment would not lead to long-term mental strain. Some patients described some kind of risk-benefit assessment weighing fighting against cancer and accepting a long-term health risk. Potentially lethal side effects downgraded the acceptability significantly. ***Medication*** beyond the adjuvant therapy were perceived differently. While some participants stated that side effects would be tolerable if treatable with drugs, others would refuse further medication (Q1.4), also because these could cause additional side effect. Similarly, ***altered blood counts*** due to the adjuvant treatment did not affect acceptability for some patients, whereas others valued such to reduce acceptability and to be concerning (Q1.5).

Patients expressed different preferences regarding the **route of administration**. A major aspect was ***being convenient and easy to integrate*** into patients’ lives. While many participants assumed pills to be simple and pleasant to take, increasing independence and autonomy (Q1.6), others worried that these would restrict their everyday life, for example because punctual time point of intake would interfere with their job schedule. Accordingly, some patients regarded appointments for infusions as easier to be integrated into everyday life than daily pill intakes (Q1.7). In general, ICI requiring only single infusions was more acceptable than cICI requiring more frequent and longer appointments. Some patients also considered appointments for infusions as a nice pastime, where they would be able to recreate or to chat with physicians, nurses or other patients. Different aspects of the therapy form also caused patients thinking about ***responsibility and control***. Some welcomed the release from responsibility when receiving infusions instead of pill intakes where patients oversee the intake as prescribed (Q1.8). Also frequent blood samplings were assumed to create a feeling of control and reliability. ***Experience of and association with disease*** also guided preferences for therapy forms. Patients having bad experiences with finding veins reported reduced acceptability when frequent blood sampling would be necessary (Q1.9) or they came up with the idea of using ports instead of infusions. Several patients associated infusions with chemotherapies or with other severe diseases. Additionally, some stated that the administration of infusion in appropriate premises would be mentally more burdensome as this would make thinking about melanoma more present (Q1.10). Other patients assumed no impact of the route of administration as side effects were seen as most important aspect (Q1.11); also blood sampling had no impact on acceptability for a range of participants (Q1.12).

Many participants did not mention **visits to physician**, which were described in the scenarios, as explanation for acceptability for a therapy, indicating ***no impact*** (Q1.13). When asked about this aspect, many stated that this would be ***reassuring*** and give a certainty to be in good hands (Q1.14). Others thought this would restrict them in planning and therefore have ***impact on everyday life*** (Q1.15). For others, regular physician visits would increase the ***presence of the disease*** and therefore be a mental burden (Q1.16). None considered whether the different types of services provided in the visits (e.g., electrocardiogram, blood sampling) would make a difference.

In the TTO questionnaire preceding the interviews, participants were asked to state the acceptable risk of recurrence and dying. In the interviews, the **prospect of treatment benefits** was described as decisive motive in the evaluation of adjuvant therapies. In the ***prospect***, many patients required a minimum level of success and showed reduced acceptability when the rate of success was too low (Q1.17). However, most of the scenarios would still be acceptable for many patients even without high increase in chances of success. Some rated almost every type of therapy as acceptable if it was a reliable prevention of recurrence or death. Nevertheless, patients were aware that there is no treatment with complete chance of recovery. The ***consequences*** were also considered. Patients weighed side-effects with the prospect of treatment benefits (Q1.18). Partially, patients regarded a therapy with severe side-effects as acceptable even with little chance of success if there was no alternative treatment or if it provided a chance to prevent a chemotherapy. In the evaluation of the ***risk of recurrence and the risk of dying***, some patients weighted both aspects similarly (Q1.19), whereas others perceived the risk of dying as more significant this is a terminal consequence: while there was no clarity about the state of health in case of a recurrence, it might be less severe than the primary tumour (Q1.20).

When being asked what could help or guide patients in their decision, patients stated as major **source of information and decision aid** their trusted ***physicians***. The advice of the physician would be decisive for most participants (Q1.21). They expected to receive good and comprehensive information from this provider to be able to follow this advice. Some mentioned that they would like to get a ***second opinion*** (Q1.22). For many participants, ***exchange*** with other patients or with people in their personal surrounding would be helpful (Q1.23). For others, ***independence*** was most important, and they preferred to get informed independently and make a decision on their own (Q1.24). Additionally, participants emphasized the importance of ***psychological support and counselling*** (Q1.25). Post-diagnostic care was considered important as regular contact with the physician would give a positive feeling. Others would demand professional psychological counselling.

Some patients came up with the idea of **alternative therapy methods**, stating that they would prefer ***less invasive options***, such as alternative healing methods, nutrition and prevention (Q1.26). These patients considered adjuvant therapy as preventive measures and preferred to try with gentle methods. Other patients thought that there was ***no alternative*** to adjuvant therapies and, in this case, would be open to try despite possible side effects (Q1.27).

### Way of living

**Table 2:** Cluster Way of living

Main category	Subcategory	Quote	#
**Everyday activities**	Stay active	“But the other things, if I was constantly sick so that I simply didn’t feel like being active anymore. Or can’t participate in anything anymore, then I’d die like an unwatered flower.” (P3, female, 59 years)	Q2.1
	Maintain independence	“So, you are no longer spontaneous and are dependent on others, or you are in need of help yourself. I had that in the background. You’re more of a burden to others.“ (P16, male, 35 years)	Q2.2
	Maintaining socially active	“So with the side effects, it’s basically also the communication with other people. So I just need […] I communicate with other people in some way all day long and I think that something like that can really drop off. And that has fatal consequences for me if it is missing over a longer period of time.“ (P3, female, 59 years)	Q2.3
**Domestic environment**	Continuing family life	“So that you still have a family connection and aren’t so isolated. (…) So it’s a kind of togetherness. (…) But even if you have to go out every few weeks and get an infusion and then you’re home again afterwards. I think that’s actually the best thing for your recovery or for your well-being. (…) Well, somehow you have to get through it together (laughs).” (P1, female, 63 years)	Q2.4
	Not wanting to become bedridden or housebound	“Then you have to say that if you lie in bed for half the day, you don’t get anything done. Not just because you have to lie in bed, but because of the multitude of things. Even if you have diarrhoea and vomiting, you can’t leave the house any more. You’re almost one hundred per cent restricted.” (P11, female, 51 years)	Q2.5
	Place of treatment	“If I’m that bad, I’m assuming that I’ll hopefully be treated in hospital and won’t have to stay at home. If I had such side effects at home and I can no longer manage my household or look after my children or I have a sick husband at home who suffers from multiple sclerosis. That will make it hard for me to bear if I’m basically a total failure here at home too.” (P9, female, 48 years)	Q2.6
**Occupation**	Compatibility with work	“Well, of course, I think I also said that taking tablets is definitely the easier option in the end. In view of the fact that I’m going to be very busy at work for a few more years and then you have to find ways of organising how you deal with it, in terms of time and organization.” (P14, male, 61 years)	Q2.7
	Feeling of being needed	“Well, it’s - I’d like to continue doing my usual everyday life, I’ll say roughly. Like going to work and because that’s actually always, yes (exhales) also good, right? It’s also good for the soul, like that. I’m going to say being needed and (…) that kind of nonsense is always difficult, of course, isn’t it? Then work also becomes a burden (laughs)“ (P4, female, 52 years)	Q2.8
**Quality of life**	Presence of quality of life	“So, the success rate in relation to the quality of life. In other words, the side effects or symptoms are not so serious. (…) Yes, I found that quite frightening and it really is a considerable intervention in the quality of life.“ (P15, female, 61 years)	Q2.9
	Life extension versus quality of life	“If you know that you can actually only prolong life a little, or prolong life sometimes means pro-long suffering. So I would, FOR ME NOW, from my current perspective, rule that out for me. (…) I believe that quality of life is a bit more decisive. Not simply living longer, but the quality, right?” (P4, female, 52 years)	Q2.10
**Patient characteristics**	Age and general mental and physical conditions	“Well, if I was 30 or something, I would have answered that very differently (…). Yes, then I would really take it upon myself, because I think the body is, at that moment you are ill, but healthier and you still have more energy and strength to get through something like that than when you are older.“ (P15, female, 61 years)	Q2.11
	Place of residence and mobility	“And of course it also depends, because I still live here in the countryside, which for me would also mean that I would probably have to go to a university clinic somehow […] it’s always associated with a long journey.” (P15, female, 61 years)	Q2.12
	Family status	“I also found that again, yes, I found it really exciting for someone who is still young but doesn’t have any children planned yet. And then suddenly they’re faced with such decisions, perhaps. I think it’s crucial (…) I think when other people perhaps say, no, I’m young and I haven’t thought about children yet anyway and now I’d rather do the treatment first, I think that’s very difficult for young, very young people to imagine the scenario and say, no, no, first of all I, no, first of all I want to be healthy.“ (P13, female, 51 years)	Q2.13
	Progression of the disease	“You really have to be a serious case in the treatment of melanoma to take that for yourself.” (P10, male, 56 years)	Q2.14

The cluster “way of living” (Table 2) described the influence that activities and circumstances of life organisation have on the acceptability of adjuvant therapies. This cluster was smaller compared to the previous but present in all 17 interviews. Patients explained that the impact on **everyday activities** would affect the acceptability of adjuvant therapies. Participants stated that they would like to ***stay active*** (Q2.1) and conduct activities such as cycling, walking, going to the gym or being able to go into nature. No longer being as efficient as before would reduce patients’ acceptability. Additionally, participants stressed that they wanted to ***maintain independence*** (Q2.2) both in terms of being able to manage their household and everyday life (e.g., cooking, gardening, body care) and in terms of being free in organising their lives. They wanted to be free in determining their way of living despite treatment and potential side effects as well as to not being a burden to others. Likewise, ***maintaining socially active*** was important to the participants as they wished to be able to keep appointments and to keep in touch with other people (Q2.3).

Similarly, participants mostly wanted to stay in their **domestic environment**. They wanted to ***continue their family life*** both in terms of their family being present throughout treatment (Q2.4) and in terms of being with the family in the long term through longer survival. They did ***not want to become bedridden or housebound***; this would make the therapy less acceptable (Q2.5). The ***place of treatment*** was important: While some wanted to be able to stay at home through outpatient treatment, one patient did not want to be treated inpatient as she did not want to experience severe side effects at home (Q2.6).

Regarding their **occupation**, patients discussed the ***compatibility with work*** of different treatments. Views differed as to whether pills and infusions would be compatible with the current job (Q2.7). Some patients emphasized that their job was important to them to pursue everyday life and to get the ***feeling of being needed*** (Q2.8).

Participants mentioned **quality of life** as important result of a treatment. For them, ***the presence of quality of life*** was critical, and scenarios would be rated as acceptable if there would still be the sufficient quality of life according to personal judgment (Q2.9). Some side effects (e.g., skin changes, gastrointestinal problems, being bedridden) were associated with reduced quality of life making treatment less acceptable. It was important to maintain a zest for life. Patients weighed ***life extension versus quality of life***. They hoped for increased quality instead of just prolonging life. A therapy would be rated as less acceptable if it would only be a life-prolonging measure that causes suffering (Q2.10).

Above this, participants assumed **patient characteristics** to have an impact on the acceptability of treatment options. Patients assumed ***age and general mental and physical conditions*** to affect acceptability. Older patients assumed that they would be more willing to accept side effects when being younger and healthier (Q2.11). Patients with certain health conditions reported that they would fear that the treatment would intensify existing diseases. ***Place of residence and mobility*** were considered relevant when treatments would require frequent visits to physicians, such as for receiving infusions (Q2.12). ***Family status*** might impact on acceptability of treatment. Patients would like to maintain their social role and would like their family as support throughout treatment. In patients with desire to have children the reduced fertility caused by the treatment might reduce acceptability (Q2.13). Also, ***progression of the disease*** might affect acceptability with patients being more likely to accept a treatment when more advanced melanoma having (Q2.14).

## Emotions and feelings

**Table 3:** Cluster Emotions and feelings

Main category	Subcategory	Quote	
**Optimism and hope**	Grasping at the last straw	“But that also has to do with the combative nature of one or the other, […] in the end I’m still grasping the straws and then I’m not yet at this level of stress for me personally so that I throw in the towel right away, no.” (P10, male, 56 years)	Q3.1
		“(laughs) Yes, I actually ticked them at random because my basic idea was that I don’t see it as a major impairment or a major restriction, but actually as a source of hope.”’ (P9, female, 48 years)	Q3.2
	Prospect of a healthy life	“Because that’s simply because I want to get well again and I don’t want to die and that’s why I rated it the same.” (P13, female, 51 years)	Q3.3
	Therapy-related aspects awakening hope and optimism	“Yes, I would actually. Because it’s just a year long. So if it was promised to be like this for many years, then I would probably go down, but I think this time limit would make it bearable for me.” (P8, female, 53 years)	Q3.4
		“Yes, it doesn’t have to be the side effects. There is always a chance that they can occur. And I’m just a bit optimistic that not everything or not so much will happen” (P12, female, 72 years)	Q3.5
**Mental strain**	Burdensome situation	“Yes, it’s really frightening for me to just lie in bed for such a long time and to have this feeling of vomiting, diarrhoea and, as I said, this concentrated package of side effects, that really scares me off. Because I could also imagine that you then slip, so I could imagine that I would absolutely slip into a depression. And I think that would really knock me out mentally” (P15, female, 61 years)	Q3.6
		“Well, let me tell you, I go in as a very sporty and fit 61-year-old and, if I may say so, I don’t think I’ll walk out of there at 62 and be almost as fit as I was on the first day. I can’t imagine that at all. You can do activities, you’re reasonably fit again, but I don’t believe that you’re going to walk around the world whistling happily like I am today” (P14, male, 61 years)	Q3.7
	Fear of side effects	“Yes, well, if, as I said, because of the changed values, if the therapy has to be discontinued for the time being and possibly, if I understood correctly, even completely […] And these are simply fears that arise as a result. (…) Exactly and that it will be stopped and, as I said, that then who knows where the journey will take me, no, how I’ll feel afterwards.” (P15, female, 61 years)	Q3.8
	Fear of disease	“Then of course you ask yourself the question: ok, would you do that now if you get the cancer again? Would you go through it again now? And then you also ask yourself, and what do you do if it doesn’t help again? Or maybe it comes back again. (…) Because if it happens again, it’s very scary, so you think about the fact that you probably wouldn’t be able to cope with it, right?” (P7, female, 37 years)	Q3.9
**Experience with diseases**	Own experiences	“Well, I know that during my treatment a few years ago, I was constantly having blood taken and constantly had a catheter because I had bladder problems and where you say, (groans) now it’s coming again and when you always know, oh, now the infusion is coming again, then blood is tak-en so, yes, it’s always a cycle where you know, oh, that’s coming again. […] So I always found that really horrible and at some point the veins are so gone (laughs) that it’s already scarred, the vein.” (P13, female, 51 years)	Q3.10
	Experiences in personal environment	“For example, my mother was also diagnosed with cancer two years ago, breast cancer. And she had to go through chemotherapy and radiation, so I accompanied her everywhere and drove with her, and, and, and. Where I thought, that’s much worse than what is basically being asked here.” (P9, female, 48 years)	Q3.11

The cluster “emotions and feelings” (Table 3) describes emotional aspects that can influence decisions about therapy. Participants expressed **optimism and hope** in the interviews. Several persons stated that they would be ***grasping at the last straw*** meaning that they would have the will to try and get through everything (Q3.1). They would accept therapy to prolong life and be fighting and not wanting to give up. The therapy options would be perceived as hope and opportunity (Q3.2). Additionally, they perceived adjuvant therapies as ***prospect of a healthy life*** and having fought cancer, as therapy could improve the state of health by combating possible side effects of malignant melanoma (Q3.3). Additionally, ***therapy-related aspects awaked hope and optimism*** for participants. On the one hand, they underlined that therapies in all presented scenarios were restricted to one year and therefore bearable due to being temporarily (Q3.4). On the other hand, they would hope for a positive course of treatment with only some of the described side effects (Q3.5). Overall, the percentage chances of success were important. Some participants also stated that they could start a treatment, and, in the event of severe side effects, they regarded discontinuing treatment as potential resort. However, others feared that they would have reached the end of therapy options in the case of severe side effects or recurrence.

Patients stated that the situation described in the TTO questionnaire would cause **mental strain** due to the ***burdensome situation*** described. They would perceive the necessity to make a therapy decision, the unclear chances of recovery and the potential side effects as mentally and emotionally stressful (Q3.6). Some also expected that the treatment would change them physically and mentally in the long-term (Q3.7). Some participants emphasized their ***fear of side effects***. On the one hand, they feared the occurrence of side effects and bad blood counts, as well as the life-threatening nature of some of the side effects. On the other hand, they were concerned that therapy would fail or would need to be discontinued due to severe side effects (Q3.8). This would cause uncertainty, fears and doubts and, at the same time, a break would extend the duration of burdensome treatment overall. However, patients expressed ***fear of disease***, such as a recurrence or spread of the melanoma (Q3.9). Due to the life-threatening nature of this disease, they would accept more severe side effects. They stated that a recurrence despite therapy would be almost unbearable.

In thinking about the scenarios, patients reflected on previous **experience with diseases**. They thought about their ***own experiences*** with cancer in the past, even though this had minor influence on assessment regarding form of administration and psycho-oncological care. Additionally, the compared descriptions in the scenarios with previous negative experiences with side effects (Q3.10). Others reported having neither medical expertise nor experience with serious illness, which is why they were afraid of the described treatment options. They also compared the scenarios with ***experiences in their personal environment***. This included both cancer and non-cancerous diseases. While some reported about cases in which no cure was possible despite therapy or negative impacts of treatment on daily life (e.g., need for regular dialysis) and long-term outcomes (e.g., impact on personality), others made comparisons to cases with positive course of a life-threatening disease in their circle of acquaintances. Despite being medically different, patients often compared treatments involving infusions with chemotherapies (Q3.11). While some considered adjuvant therapy as positive as it would be less burdensome than chemotherapies, others suspected similarities between adjuvant treatments and chemotherapies.

## Discussion

Adjuvant therapies can be associated with significant toxicity, which can lead to chronic side effects, particularly in the case of immunotherapy. When making treatment decisions, patients and physicians must weigh the benefits and risks of available therapy options. Ultimately, the decision must be made by the patients themselves. To advise patients appropriately, it is crucial to understand how they assess toxicity and what other parameters are meaningful to them.

Our findings suggest that patients consider the prospect of treatment benefit to be an important factor in making treatment decisions. This means that therapies are expected to improve patient outcomes at least minimally, so that associated costs and risk are accepted. Severe side effects reduce acceptability of adjuvant treatment (Lodde et al. 2021; Kähler et al. 2023), but our study shows that patients would be willing to accept severe and persistent side effects to improve their health status and prolong their lives. Many patients in our study rated OS and DFS similarly, demonstrating that living without recurrence is relevant to patients even though recurrence is usually not noticeable to patients. Nevertheless, it is desirable for patients to avoid recurrences, as these indicate a poor and possibly fatal outcome. This supports DFS and distant metastasis free survival, in addition to OS, can be used as meaningful endpoints in clinical research.

There were differences in the preferred route of administration. While some patients emphasised the independence of tablet therapy, others assumed that recurring physician’s appointments would be easier to integrate into their daily routine than being tied to strict times of taking tablets. A heterogeneous picture also emerges from quantitative studies of preferred route of administration (Weilandt et al. 2020; Kähler et al. 2023). Other studies have shown that when evaluating adjuvant therapy, participants attach less importance to the route of administration and prioritise aspects such as OS (Liu et al. 2019; Stenehjem et al. 2019). Additionally, our study shows that convenience is highly valued in evaluating a therapy regimen, an aspect that is rarely investigated in quantitative treatment preference studies.

In addition to compatibility with daily life, patients evaluate the therapy according to its impact on HRQoL; side effects with a negative impact on HRQoL were rated as particularly burdensome. These include immunotherapy-induced changes in skin appearance induced due to its visibility. Patients were also concerned that gastrointestinal problems, tiredness and weakness would limit their ability to be active. A previous study showed that HRQoL restrictions were the third most common reason for treatment discontinuation (Lodde et al. 2021).

The decision about adjuvant treatment is a highly emotional one for patients. Participants expressed a feeling of ‘grasping at the last straw’. Accordingly, patients would not base their decision solely on objective parameters such as OS and RFS; they would not want to give up prematurely, but would want to feel that they have tried everything. Above this, our results show the worry and anxiety that the scenarios cause patients due to the potential for life-threatening melanoma, life-threatening side effects and poor blood counts leading to unpredictable long-term effects. Aspects that gave participants a sense of control were reassuring, such as the possibility of pausing or stopping treatment if side effects would become too severe and regular physician visits. The described emotional impact of such decisions leads participants in our study to suggest psycho-oncological care. They criticise that they have not received sufficient emotional support in the past. This aligns with previous research showing that one third of patients do not feel adequately informed or supported (Lehmann et al. 2009).

Besides psycho-oncological support, patients underline the importance of advice and support during their physician visits. Communication with their treating physician was seen as a crucial aspect in the decision-making process. Receiving inadequate or contradictory information can increase in anxiety about cancer and lead to decision-making conflicts (Faller et al. 2016). In contrast, studies show that good communication between physician and patient leads to greater satisfaction, better disease management and greater satisfaction (Mager and Andrykowski 2002; Schofield et al. 2003; Fallowfield and Jenkins 2004; Hack et al. 2005; Lehmann et al. 2009). Patients who feel adequately informed by good physician-patient communication show increased compliance, which in turn has a positive effect on the treatment success (Lehmann et al. 2009). It is important for clinicians to understand their patients’ communication preferences and needs and to agree on treatment goals with them to avoid misunderstandings and conflicts (Lehmann et al. 2009). This supports the importance of physician-patient communication, which is emphasized in the S3 guidelines for the treatment of malignant melanoma (Deutsche Krebsgesellschaft, Deutsche Krebshilfe, AWMF 2020).

Participants in our study assumed that younger patients would be more willing to accept side effects than older patients. This is consistent with quantitative data showing that older patients demand higher effectiveness of a treatment (Kähler et al. 2016, 2023). In another study, older patients were less likely to start an adjuvant treatment (Lodde et al. 2021). Weilandt et al. (2021) describe that with increasing age, patients prioritize toxicity and impact on daily life over effectiveness when evaluating treatment options.

This study is one of the first to qualitatively explore patients’ willingness to accept adjuvant therapy in melanoma and its toxicity, providing a deeper insight into what is important to individuals faced with such critical decisions. The value of qualitative research in this context has previously been highlighted by a systematic review which noted that “one difficulty with hypothetical scenarios is that patients do not know what they have not experienced” (Livingstone et al. 2020), a shortcoming of hypothetical study designs that was also mentioned by participants in our interviews (“*All with the proviso that if I am in the specific situation […] then I may also think quite differently about it and say I’ll grasp at any straw*”, P14, w, 61). It was important for the participants to be able to talk about what they had read and answered in the quantitative questionnaire. This is also demonstrated by the 100% participation rate of all patients contacted, which shows that it was important for participants to talk about the questionnaire and their answers. Even though participants were not prompted to think of their previous melanoma, they stated that completing the questionnaire made them to think intensively about this experience and brought back memories (“*The problem was that all the unpleasant feelings or this shock basically came back*” P3, w, 59). It needs to be acknowledged that these memories might be affected by recall bias in that current emotions and circumstances may have affected participants’ responses. The results of this qualitative study can be useful for clinical communication and for future quantitative research, as hypothetical treatment scenarios can be revised according to these results. However, in this qualitative study it must be acknowledged that (for ethical reasons) none of the participants was in the actual situation of making decisions about adjuvant therapies. This may lead to a more realistic response to the scenarios, but decision-making is still hypothetical. So, the decision might be different in a real situation; patients in our interview said they thought the acceptability would probably be higher.

Participants were recruited at a single study center. Due to the possibility that patients at this study center may not be representative of patients in Germany, applicability of our results to broader patient populations needs to be confirmed. The majority of the participants were female. However, neither our qualitative results nor quantitative studies (Lodde et al. 2021; Kähler et al. 2023) indicated gender differences in terms of treatment preferences.

Although coding of the interviews was carried out by one researcher, regular team meetings were held in which codes and categories were discussed in detail, ensuring the trustworthiness of the resulting category system.

In conclusion, our study shows that patients are very willing to undergo adjuvant therapy, even when facing toxicity, although the assessment of potential side effects and prospects of treatment benefit is highly individual. Therefore, it is important to consider personal patient preferences to make appropriate and shared decisions. Understanding the increasing impact of the patient perspective, especially in drug approval processes, underscores the importance of incorporating both qualitative and quantitative analyses. This approach facilitates a more comprehensive understanding of the patient experience, providing valuable insights for decision-making and potential application in clinical trials.

## Data Availability

The datasets generated during and/or analysed during the current study are available from the corresponding author on reasonable request.
